# Single-Port Laparoscopic Surgery Is Feasible and Safe for Hepatic Left Lateral Sectionectomy for Benign Liver Lesions

**DOI:** 10.1155/2019/1570796

**Published:** 2019-07-01

**Authors:** Yuan Cheng, Ze-sheng Jiang, Xiao-ping Xu, Wen-fa Huang, Guo-lin He, Chen-jie Zhou, Jia-sheng Qin, Yi Gao, Ming-xin Pan

**Affiliations:** ^1^Department of Hepatobiliary Surgery, Zhujiang Hospital, Southern Medical University, Guangzhou, Guangdong Province, China; ^2^Second College of Clinical Medicine, Zhujiang Hospital, Southern Medical University, Guangzhou, Guangdong Province, China

## Abstract

**Background and Objectives:**

The feasibility and safety of single-port laparoscopic surgery for left lateral liver lobectomy are largely unknown. This study is aimed at comparing the effectiveness and safety between single-port laparoscopic (SPL) and conventional multiport laparoscopic (CL) surgeries for hepatic left lateral sectionectomy.

**Methods:**

A total of 65 patients receiving laparoscopic hepatic left lateral sectionectomy between January 2008 and July 2015 were included and divided into the SPL group (*n* = 40) and the CL group (*n* = 25).

**Results:**

There was no significant difference in the operative time, estimated intraoperative blood loss, length of hospital stay, and incidences of postoperative complications (biliary leakage, hemorrhage, and contusion at incision) between groups (all *P* > 0.05). However, the SPL group had a significantly lower VAS pain score (at 24 h but not 7 days postoperation) and higher cosmetic satisfaction scores (at both 2 months and 6 months postoperation) than the CL group (all *P* < 0.01). Moreover, multivariate linear regression analysis further confirmed the superior pain score and cosmetic outcome in the SPL group.

**Conclusions:**

Single-port laparoscopic hepatic left lateral sectionectomy is a safe and feasible treatment for patients with lesions in the left hepatic lobe. Patients with benign lesions in the left hepatic lobe are more suitable to receive single-port laparoscopic hepatic left lateral sectionectomy than those with malignancies.

## 1. Introduction

Laparoscopic hepatectomy is a well-recognized and standard treatment for liver diseases [1]. Currently, laparoscopic surgery has become the standard treatment for left lateral hepatic lobe disease [2]. Single-port laparoscopic surgery was firstly developed in 1992 [3], which possesses several advantages over the conventional multiport laparoscopic surgery, such as reduced postoperative pain, better cosmetic outcomes, and shorter hospital stay times [4–6]. Single-port laparoscopic surgery has been widely used in several abdominal operations in the fields of gastroenterology, hepatology, obstetrics, gynecology, and urology [7–11].

The first laparoscopic “left lateral hepatic segmentectomy” was published in1996 by Azagra et al. in surgical endoscopy [12]. However, there is no cohort study on single-port laparoscopic hepatic lobectomy. In addition, the effectiveness and safety of single-port laparoscopic hepatic lobectomy remain to be evaluated. We have designed a five-step single-port laparoscopic hepatic left lateral sectionectomy technique based on the anatomical features of the left lateral hepatic lobe and started to perform this surgery in our hospital since July 2012. Therefore, the purpose of this study was to report the effectiveness and safety of the single-port laparoscopic hepatic left lateral sectionectomy.

## 2. Methods

### 2.1. Patients

A total of 65 patients receiving laparoscopic hepatic left lateral sectionectomy in our hospital between January 2008 and July 2015 were included. The five-step single-port laparoscopic hepatic left lateral sectionectomy was designed and performed in July 2012 in our hospital. After which, multiport or single-port laparoscopic surgery was performed according to patient's preference. Among the 65 included patients, there were 40 patients receiving single-port laparoscopic hepatectomy (SPL group) and 25 cases receiving the multiport laparoscopic hepatectomy (CL group).

The inclusion criteria included (1) lesions in the second or third segment, such as hepatolithiasis with complicating liver atrophy, symptomatic benign lesions bigger than 10 cm, or malignant tumors small than 5 cm; (2) Child-Pugh class A or B; and (3) patients who asked for the single-port or multiport laparoscopic hepatic left lateral sectionectomy. The exclusion criteria were (1) patients who refused to undergo laparoscopic surgery, (2) patients with a history of upper abdominal surgery, (3) patient who cannot tolerate laparoscopic surgery, and (4) body mass index greater than 35 kg/m^2^. This study was approved by the Ethics Committee of Zhujiang Hospital, Southern Medical University, Guangzhou, Guangdong Province, China (No. ZJYY-201 5-GDEK-001). Patients and their family members approved all surgical procedures and signed the informed consent form.

### 2.2. Data Collection

Patient's demographic and clinical characteristics including age, sex, weight, and types of lesion were collected. The intraoperative data included total blood loss and operative duration. The postoperative outcomes included the length of hospital stay, postoperative complications (biliary leakage, bleeding, and incision infection), pain scores, and cosmetic outcome. Postoperative pain scores were assessed at 24 hours and 7 days postoperation according to the standard visual analog scale (VAS) (range, 0 (no pain) to 10 (maximum pain)). At 2-month and 6-month follow-up, the patients were asked to self-rate their own cosmetic outcome of the surgical incision by a questionnaire as follows: 1 (very dissatisfied: change in abdominal appearance was significant and unacceptable), 2 (acceptable: obvious change in abdominal appearance but still acceptable), 3 (satisfied: slight change in abdominal appearance), and 4 (very satisfied: almost no change in abdominal appearance).

### 2.3. Surgical Procedure

All the surgeries in this study were performed by the same surgical team. The patient was placed in the French position, and the surgeon stood between the patient's legs. The first assistant who held a laparoscope stood at the right side of the patient, and another assistant stood at the left side of the patient to assist in exposing the surgical field. The carbon dioxide pneumoperitoneum was maintained at 13 mmHg.

**Figure 1:**
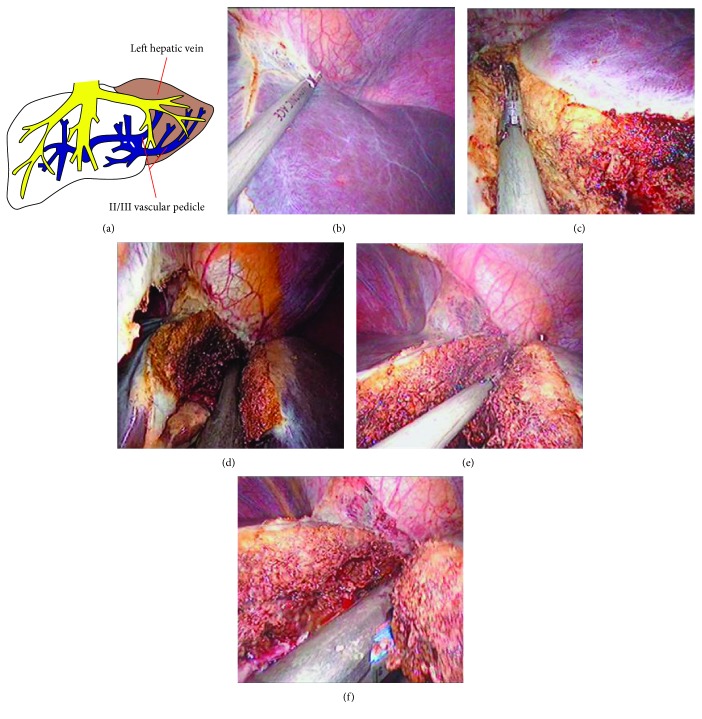
(a) The “five-step” single-port laparoscopic hepatic left lateral sectionectomy is developed based on the anatomical features of the hepatic left lateral lobe. (b) The left hepatic lateral lobe was fully freed. (c) The ultrasonic scalpel was used to expose the vascular pedicle of segments II and III. (d) Articulating linear cutter stapler was used to divide the II/III vascular pedicle. (e) The ultrasonic scalpel was used to expose the left hepatic vein. (f) Linear cutter stapler was used to divide the left hepatic vein.

In the single-port laparoscopic surgery, all operations were performed using a custom-made “hand-shaped” single-port trocar through a 2.5 cm umbilical incision. The five-step operation was performed as follows (Figure 1):

1. A 30° laparoscope (Olympus, Tokyo, Japan), a grasper (Kanger, Tonglu, China), and ultrasonic scalpel (Ethicon Endo-Surgery, Cincinnati, OH, USA) were placed into the abdominal cavity. After abdominal exploration, the perihepatic ligaments and the round, falciform, left coronary, left triangular, and hepatogastric ligaments were sequentially divided by an ultrasonic scalpel to fully expose the left hepatic lateral lobe (Figure 1(b))
2. Based on the preoperative imaging examination, the positions of large intrahepatic vessels were identified. The ultrasonic scalpel was then used to dissect the hepatic parenchyma anterior or superior to the vascular pedicle of segments II and III (Figure 1(c))
3. The II/III vascular pedicle along with a little parenchyma was transected using an articulating linear cutter stapler (Ethicon Endo-Surgery, Cincinnati, OH, USA) through the left 12 mm main operating port (Figure 1(d))
4. Based on the preoperative imaging examination, an ultrasonic scalpel was used to dissect the hepatic parenchyma anterior, superior, or inferior to the left hepatic vein (Figure 1(e))
5. Linear cutter stapler (endoclip) was used to transect and seal the left hepatic vein along with the surrounding liver tissue. The hepatectomy was completed (Figure 1(f))

At the last step, the bipolar electrocautery tool was used to coagulate the hepatic wound bed. The suture was used to stop excessive bleeding if necessary, and a normal saline solution was used to flush the hepatic cutting surface. The resected benign lesion specimens were placed in a plastic bag (Covidien, Mansfield, OH, USA) and externalized after fragmentation. The resected malignant lesions were removed through the incision of the umbilical port by expanding the port size. A latex drainage tube was positioned close to the cutting surface of the liver and was introduced out through the umbilical incision. All incisions were closed with intradermal sutures, and a diluted ropivacaine solution was injected in the surrounding tissue of the incision or the port site.

A four-port procedure was used in the conventional laparoscopic hepatic left lateral sectionectomy. First, a 30° laparoscope was introduced into the abdominal cavity through the 1 cm umbilical incision. A 12 mm or 5 mm trocar was inserted under the left costal margin at the pararectal line and under the right costal margin at the pararectal line, respectively. An incision was then made in the left costal margin at the anterior axillary line, and a 5 mm trocar was inserted to expose the surgical field. The detailed protocol was the same as that in the single-port laparoscopic hepatic left lateral sectionectomy.

### 2.4. Statistical Analysis

Continuous data were expressed as the mean ± standard deviation (SD) and compared by Student's independent *t*-test or Mann-Whitney *U* test if normality was not assumed. The operative time was used to represent the learning curve. Categorical data were presented as number and percentage (%) and were tested by Chi-square test or Fisher's exact test if any expected value ≤ 5 was observed. Simple and multiple regression models were used to investigate the group difference of the VAS pain score and the cosmetic score while other covariates were controlled. The statistical significance level for all the tests was set at a *P* value < 0.05. All analyses were performed using IBM SPSS version 20 (SPSS Statistics V20, IBM Corporation, Somers, New York).

## 3. Results

### 3.1. Patient's Demographic and Clinical Characteristics

A total of 65 patients were analyzed, including 40 patients receiving single-port laparoscopic hepatectomy (SPL group) and 25 cases receiving the conventional laparoscopic hepatectomy (CL group). The mean age for all patients was 42.55 ± 9.31 years. The major indication for hepatectomy was cavernous hemangioma (67.7%), followed by hepatocellular carcinoma (HCC) (21.5%), focal nodular hyperplasia (FNH) (9.2%), and perivascular epithelioid cell tumors (PEComas) (1.5%). As shown in Table 1, there was no significant difference in patient's age, BMI, indications for hepatectomy, Child-Pugh score, tumor size, and lesion size (all *P* > 0.05), indicating that the two groups were comparable.

### 3.2. Intraoperative Results

The Pringle maneuver was not used in this study. All the operations were successfully carried out except for one FNH patient in the SPL group which was switched to multiport laparoscopic surgery due to intraoperative bleeding when the left hepatic vein was not completely closed by a linear cutter stapler. There was no significant difference in the operative time (100.50 ± 21.83 vs. 89.04 ± 21.70 min, *P* > 0.05, Table 2) and the estimated blood loss (149.00 ± 109.26 mL vs. 139.00 ± 96.07 mL, *P* > 0.05, Table 2) between groups.

### 3.3. Postoperative Outcomes

The postoperative outcomes were compared between groups in Table 2. The length of hospital stay was not different between the SPL group and the CL group (4.60 ± 1.52 vs. 4.28 ± 1.59 days, *P* = 0.225). As for the postoperative complications, there were no deaths in both groups. One patient in the SPL group had bleeding on the hepatic cutting surface (the daily blood loss = 200 − 300 mL, lasting for 3 days), and one patient in the CL group had biliary leakage (the daily loss = 50 − 200 mL, lasting for 15 days); both of which were resolved by conservative treatments. No infection was reported among all patients. There was no significant difference in the incidences of biliary leakage, hemorrhage, and contusion at incision between groups (all *P* > 0.05).

The VAS pain score at 24 h postoperation was significantly lower in the SPL group than in the CL group (1.63 ± 0.67 vs. 2.63 ± 0.95, *P* = 0.002). However, no significant difference was observed at 7 days postoperation between groups (*P* = 0.227). The postoperative pain in the CL group was mainly caused in the regions around the subumbilical and subxiphoid ports. Cosmetic satisfaction scores were higher in the SPL group than in the CL group at both 2 months (3.60 ± 0.50 vs. 2.80 ± 0.50, *P* < 0.001) and 6 months postoperation (3.75 ± 0.44 vs. 3.08 ± 0.49, *P* < 0.001).

All patients were followed up at outpatient clinics as scheduled, and no patient was lost to follow-up. No disease recurrence or residual was observed during the follow-up period.

### 3.4. Linear Regression Analysis

To further confirm the differences in the VAS pain score and the cosmetic score between two groups, multivariate linear regression analyses were performed. The results of the VAS pain score were shown in Table 3. In the multivariate linear regression model adjusted for other clinical confounding factors, the CL group had a significantly higher VAS score than the SPL group at 24 hours postoperation (*B* = 0.68, 95%CI = 0.22 to 1.14; *P* = ‐0.005). Nevertheless, there was no significant difference at 7 days postoperation (*P* > 0.05).

Table 4 demonstrated the results of the cosmetic score. Consistently, the CL group had a lower estimated cosmetic score than the SPL group at both 2 months and 6 months postoperation (*B* = ‐0.87 and -0.63, 95%CI = ‐1.14 to -0.59 and -0.89 to -0.37, respectively; both *P* < 0.001).

## 4. Discussion

In this study, we compared the effectiveness and safety between single-port laparoscopic and multiport laparoscopic surgeries for hepatic left lateral sectionectomy. The results showed that there was no significant difference in the operative time, intraoperative blood loss, length of hospital stay, and the incidences of postoperative complications between groups. However, the SPL group had a significantly lower VAS pain score at 24 hours and higher cosmetic satisfaction scores at both 2 and 6 months postoperation as compared with the CL group. Moreover, multivariate linear regression analysis consistently showed that the CL group had a significantly lower VAS score at 24 hours and a higher estimated cosmetic score than the SPL group at both 2 months and 6 months postoperation. Taken together, these results suggested that single-port laparoscopic left lateral sectionectomy possesses comparable effectiveness and safety with the multiport laparoscopic surgery and can effectively reduce the postoperative pain and improve the cosmetic outcome.

A case-matched analysis by Aldrighetti et al. shows that single-port laparoscopic and conventional laparoscopic surgeries have a similar length of surgery, blood loss, postoperative complications, and length of postoperative stay in hepatic left lateral sectionectomy [13]. Likewise, Hu et al. have conducted a prospective, randomized, and controlled study on single-port laparoscopic and conventional laparoscopic surgeries for hepatic left lateral sectionectomy and found that there is no significant difference in the operative time, blood loss, and postoperative complications [14]. Consistent with these findings, our study showed that there was no significant difference in the operative times, blood loss, incidences of postoperative complications, and length of hospitalization between groups, suggesting that single-port laparoscopic surgery is feasible and safe for hepatic left lateral sectionectomy. Although previous studies had already demonstrated that single-port laparoscopic left lateral liver sectionectomy is as safe and feasible as multiport laparoscopy, the number of surgical procedures is relatively limited and the surgical instrument and procedure have not been standardized. We had tried a variety of methods to establish a standardized single-port laparoscopic left lateral liver sectionectomy and found that the custom-made “hand-shaped” single-port trocar can minimize the mutual interference between the surgical instruments. Combining with the standardized five-step operation, the surgical difficulty is not markedly elevated as compared with the conventional laparoscopic left lateral liver sectionectomy, which is easy to promote.

The main benefits of single-port laparoscopic surgery over conventional laparoscopic surgery are better postoperative pain control and cosmetic outcome [15]. In this study, the SPL group had a lower VAS pain score at 24 hours but not 7 days postoperation. This finding indicated that single-port laparoscopic hepatic left lateral sectionectomy is superior to conventional laparoscopic lobectomy in short-term pain control, which is consistent with previous studies [4, 6, 16]. In addition, the SPL group had higher cosmetic satisfaction scores at both 2 months and 6 months postoperation, exhibiting the advantage of single-port laparoscopic surgery in cosmetic outcomes.

The consensus group acknowledged that indications for laparoscopic liver resection should not extend to benign liver diseases [17]. However, there are still a few studies reporting laparoscopic liver resection for benign liver diseases [14, 18–21]. Most of the literature indicates that the cases of benign liver diseases for laparoscopic surgery should be appropriately selected and the operation should be performed by experienced surgeons. Among the 40 cases in the SPL group, there were 33 patients with benign diseases and 7 cases with malignancies. At the early stage, we selected both benign and malignant liver diseases for the single-port laparoscopic surgery. The resected benign specimens can be removed after fragmentation, and patients had a satisfactory cosmetic outcome. By contrast, in the patients with malignancies, the umbilical port should be enlarged to keep the integrity of the resected tumor tissue, thus compromising the cosmetic outcomes. Therefore, multiport laparoscopic surgery or open surgery was gradually adopted for malignant hepatic tumors in our hospital. Hence, we believe that compared to cancer patients, patients with benign lesions in the left hepatic lobe are more suitable to receive single-port laparoscopic hepatic left lateral sectionectomy to achieve a better cosmetic outcome. The cases of benign liver diseases receiving single-port laparoscopic surgery were rigorously screened in our patients, including those with symptomatic benign lesions or benign liver diseases with a risk of malignant transformation.

**Figure 2:**
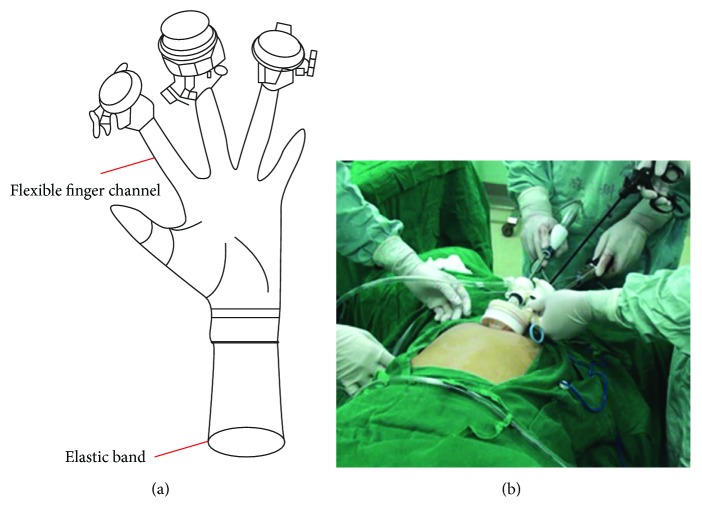
The device used in single-port laparoscopic hepatic left lateral sectionectomy. (a) The “glove-shape” trocar with fingers, allowing laparoscopy and different instruments to pass through the umbilical port simultaneously during operation (b).

**Figure 3:**
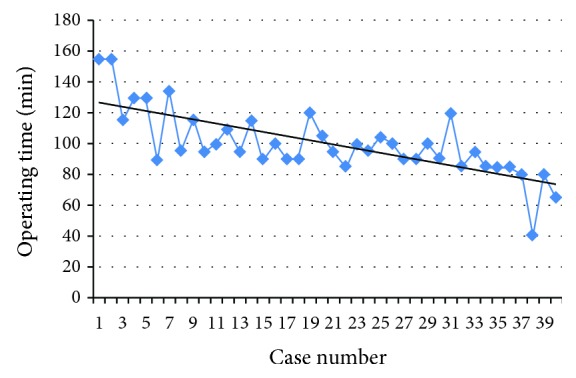
The change of operative time of single-port laparoscopic hepatic left lateral sectionectomy performed in this study.

Hepatic left lateral sectionectomy can be performed via the umbilical port due to the unique anatomical features. The predicted cutline in hepatic left lateral sectionectomy and the umbilical incision are located in the same sagittal plane, which makes it easier to dissect the hepatic parenchyma via the umbilical incision. In addition, the major vessels, left hepatic vein and vascular pedicle of segment II/III, are relatively isolated in anatomical position and can be effectively exposed and dissected. Pre- and intraoperative imaging examinations should be conducted to measure the distance from the major vessels to the diaphragmatic surface of the liver and the distance between the left hepatic vein and the vascular pedicle. These imaging data are necessary for avoiding damage to the intrahepatic vessels. Nevertheless, we can gradually skip the intraoperative ultrasound examination after familiarization with these data. To overcome the obstacles encountered in the single-port laparoscopic hepatectomy, a custom-made “glove-shape” access port was used (Figure 2(a)). This device uses a medical glove outside the abdomen to ensure pneumoperitoneum, with part of the glove fixed in the intra-abdominal cavity by an elastic band. The design of flexible fingers allows the surgeon to flexibly operate multiple instruments simultaneously. With cumulative experience, the operative time can be gradually decreased from 121.5 ± 23.69 min in the first 10 cases to 82 ± 20.30 min in the last 10 cases (Figure 3). The operative time was similar between the SPL and CL groups in our study. In addition, the operative time for single-port laparoscopic hepatic left lateral sectionectomy was shorter than that in previous studies [13, 14, 22]. Therefore, the “five-step” protocol described in this study can effectively reduce the surgical difficulty and boost the surgeon's confidence, which plays an important role in the completion of single-port laparoscopic surgery [23].

There are still some limitations of this study. First, this was a retrospective study with relatively small sample size. The pain score might be interfered since the patients were told the operative details and chose the operative approach by themselves. Moreover, between 2008 and 2015, only 25 patients were included in the CL group, which could lead to assessor and performance bias. In our hospital, we started to perform conventional laparoscopic liver resection since 2002 and have performed many cases of multiport laparoscopic hepatic left lateral sectionectomy with skilled surgical technique. After July 2012, the five-step single-port laparoscopic hepatic left lateral sectionectomy has been established and adopted in our hospital and patients choosing conventional multiport laparoscopic surgery were gradually reduced. To reduce bias, we only included the patients receiving multiport laparoscopic hepatic left lateral sectionectomy after 2008. A prospective randomized and controlled trial should be conducted to further validate the findings of this study.

## 5. Conclusion

In summary, our study showed that single-port laparoscopic hepatic left lateral sectionectomy is a safe and feasible treatment for patients with lesions in the left hepatic lobe and is superior to multiport laparoscopic lobectomy in the postoperative pain control and cosmetic outcome. Patients with benign lesions in the left hepatic lobe are more suitable to receive single-port laparoscopic hepatic left lateral sectionectomy as compared with those with malignancies.

## Figures and Tables

**Table 1:** Patient's demographic and clinical characteristics.

Parameters	SPL (*n* = 40)	CL (*n* = 25)	*P*
Age (year)	42.15 ± 10.81	43.20 ± 6.36	0.260
Sex			0.294
Male	11 (27.5)	10 (40.0)	
Female	29 (72.5)	15 (60.0)	
BMI (kg/m^2^)	24.3 ± 4.3	25.1 ± 6.5	0.608
Diagnosis			0.335
Cavernous hemangioma	29 (72.5)	15 (60.0)	
HCC	6 (15.0)	8 (32.0)	
FNH	4 (10.0)	2 (8.0)	
PEComas	1 (2.5)	0 (0.0)	
Child-Pugh score			1.000
A	40 (100.0)	25 (100.0)	
Tumor size (cm^2^)	44.10 ± 27.20	36.05 ± 25.20	0.241
Lesion size (cm)	7.4 ± 2.5	6.8 ± 2.9	0.589

SPL = single-port laparoscopic hepatectomy; CL = conventional laparoscopic hepatectomy; BMI = body mass index; FNH = focal nodular hyperplasia; HCC = hepatocellular carcinoma; PEComas = perivascular epithelioid cell tumors.

**Table 2:** Patient's operative details and outcomes.

Parameters	SPL (*n* = 40)	CL (*n* = 25)	*P*
Pringle maneuver (number)	0	0	1.000
Length of surgery (minute)	100.50 ± 21.83	89.04 ± 21.70	0.171
Estimated blood loss (mL)	149.00 ± 109.26	139.00 ± 96.07	0.984
Flatus after surgery (day)	1.90 ± 0.74	1.72 ± 0.68	0.358
Postoperative hospitalization (day)	4.60 ± 1.52	4.28 ± 1.59	0.225
Complication			
Contusion at incision	8 (20.0)	3 (12.0)	0.403
Hemorrhage	1 (2.5)	0 (0.0)	1.000
Biliary leakage	0 (0.0)	1 (4.0)	1.000
VAS (1-10)			
24 hours after surgery	1.63 ± 0.67	2.36 ± 0.95	0.002
7 days after surgery	0.68 ± 0.80	0.88 ± 0.78	0.227
Cosmetic score			
2 months after surgery	3.60 ± 0.50	2.80 ± 0.50	<0.001
6 months after surgery	3.75 ± 0.44	3.08 ± 0.49	<0.001

SPL = single-port laparoscopic hepatectomy; CL = conventional laparoscopic hepatectomy; VAS = visual analog score.

**Table 3:** VAS pain score compared between groups while covariates were controlled.

Parameters	24 hours after surgery		7 days after surgery	
	*B* (95% CI)	*P*	*B* (95% CI)	*P*
Group				
SPL	Ref.	—	Ref.	—
CL	0.68 (0.22-1.14)	0.005	0.17 (-0.29-0.63)	0.469
Sex				
Male	Ref.	—	Ref.	—
Female	-0.08 (-0.59-0.42)	0.738	0.31 (-0.20-0.81)	0.227
Age (year)	-0.03 (-0.06-0.00)	0.066	0.00 (-0.03-0.03)	0.900
Diagnosis				
Cavernous hemangioma	Ref.		Ref.	—
HCC	0.51 (-0.22-1.25)	0.166	0.04 (-0.70-0.77)	0.918
FNH	-0.38 (-1.18-0.42)	0.349	0.18 (-0.62-0.99)	0.648
PEComas	0.19 (-1.55-1.93)	0.827	0.39 (-1.35-2.13)	0.654
Tumor size (cm^2^)	0.00 (-0.01-0.01)	0.348	0.00 (-0.01-0.01)	0. 635

The multivariate linear regression model was adjusted for the demographic and clinical characteristics as well as the independent variables with significant difference between the SPL and CL groups. To eliminate the impact of the learning curve for SPL, operative time was included as a confounding factor into the multivariate linear regression model. VAS = visual analogue scale; *B* = regression coefficient; SPL = single-port laparoscopic hepatectomy; CL = conventional laparoscopic hepatectomy; FNH = focal nodular hyperplasia; HCC = hepatocellular carcinoma; PEComas = perivascular epithelioid cell tumors.

**Table 4:** Cosmetic score compared between groups while covariates were controlled.

Parameters	2 months after surgery		6 months after surgery	
	*B* (95% CI)	*P*	*B* (95% CI)	*P*
Group				
SPL	Ref.	—	Ref.	—
CL	-0.87 (-1.14--0.59)	<0.001	-0.63 (-0.89--0.37)	<0.001
Sex				
Male	Ref.	—	Ref.	—
Female	-0.01 (-0.31-0.29)	0.938	-0.01 (-0.29-0.27)	0.943
Age (year)	0.00 (-0.02-0.02)	0.872	0.01 (-0.01-0.03)	0.297
Diagnosis				
Cavernous hemangioma	Ref.	—	Ref.	—
HCC	0.31 (-0.12-0.75)	0.157	-0.03 (-0.44-0.39)	0.897
FNH	-0.23 (-0.71-0.24)	0.326	-0.28 (-0.74-0.17)	0.213
PEComas	0.61 (-0.42-1.64)	0.242	0.37 (-0.61-1.36)	0.449
Tumor size (cm^2^)	0.00 (0.00-0.01)	0.237	0.00 (0.00-0.01)	0.534

The multivariate linear regression model was adjusted for the demographic and clinical characteristics as well as the independent variables with significant difference between the SPL and CL groups. To eliminate the effect of the learning curve for SPL, operative time was included as a confounding factor into the multivariate linear regression model. *B* = regression coefficient; SPL = single-port laparoscopic hepatectomy; CL = conventional laparoscopic hepatectomy; FNH = focal nodular hyperplasia; HCC = hepatocellular carcinoma; PEComas = perivascular epithelioid cell tumors.

## Data Availability

The data used to support the findings of this study are included within the article.
